# Pain Services in the Netherlands: A Cross‐Sectional Questionnaire Study on Organisation, Tasks and Responsibilities

**DOI:** 10.1111/jan.70097

**Published:** 2025-07-21

**Authors:** Marion Giesberts, Elif Çelik, Jacqueline Van Dijk, Remko Ter Riet, Sandra Van Den Heuvel, Rianne Van Boekel

**Affiliations:** ^1^ Department of Anesthesiology, Pain and Palliative Medicine Radboud University Medical Center Nijmegen the Netherlands; ^2^ Research Department of Emergency and Critical Care, School of Health Studies, Knowledge Centre of Sustainable Healthcare HAN University of Applied Sciences the Netherlands; ^3^ Department of Anesthesiology, Pain Clinic University Medical Center Utrecht Utrecht the Netherlands; ^4^ Department of Anesthesiology and Pain (Nocepta) Ziekenhuisgroep Twente Almelo‐Hengelo the Netherlands

**Keywords:** acute pain service, pain management, pain nurse, transitional pain service

## Abstract

**Aims:**

Providing an overview of the organisation, tasks, and responsibilities of acute and transitional pain services in the Netherlands.

**Design:**

Cross‐sectional questionnaire study.

**Methods:**

An online questionnaire was sent to representatives of Dutch hospital pain services performing inpatient surgery. It included items on organisation, staffing, education, roles, tasks and responsibilities. Data were analysed descriptively.

**Results:**

Of the surveyed hospitals, 92.2% reported having an acute pain service, while only 6.5% had a transitional pain service. Most pain services (acute pain services 76.3%, transitional pain services 80.0%) are part of the anaesthesiology department. Staffing includes anaesthesiologists, nurses, and/or nurse anaesthetists, with or without pain specialisation. Acute pain service teams monitor complex pain management techniques. Nearly all acute pain services (89.8%) provide pain management training, and 60% monitor hospital‐wide pain management quality. All transitional pain services monitored opioid use post‐discharge and conducted follow‐up calls with patients.

**Conclusions:**

Acute pain services are well established in Dutch hospitals, whereas transitional pain services remain limited. Organisational structures, tasks, and responsibilities vary, with key challenges in staffing, service organisation, and education. Future research should focus on optimising staffing, expanding transitional pain services, the role of the pain nurse, and establishing a national pain management education framework.

**Implications for the Profession and Patient Care:**

This study highlights the significant impact of pain nurses as a central professional within the interdisciplinary team, contributing to quality care and education, ultimately benefiting patients.

**Impact:**

This study provides a current overview of pain services in the Netherlands, supporting pain nurses in innovating pain services, highlighting key challenges and opportunities for improvement.

**Reporting Method:**

STROBE checklist.

**Patient/Public Involvement:**

None.

**Summary**

- Provides a current overview of Dutch pain services.
- Emphasises the need for national standards in pain education.
- Highlights limited service availability and scope by staffing shortages.

## 1 Introduction

Postoperative pain remains a significant issue, with moderate to severe pain affecting 30%–80% of patients globally (PAIN OUT Research Group 2022). Severe pain increases the risks of complications, prolonged hospital stays, reduced quality of life, and chronic pain (Gan 2017). About 10%–50% of patients develop chronic pain, which leads to long‐term opioid use in 6%–35% (Schug and Bruce 2017). In turn, these problems also lead to an increased risk of complications, delayed recovery, prolonged admission duration (Gan 2017) and also have a major impact on the patient and society, such as decline in work ability, reduction in social participation and an increase in medical consumption (Baumann et al. 2023). In turn, these problems also lead to an increased risk of complications, delayed recovery, prolonged admission duration (Gan 2017) and also has a major impact on the patient and society, such as decline in work ability, reduction in social participation and an increase in medical consumption (Baumann et al. 2023). Patients with limited health literacy are at increased risk of these consequences (Kim et al. 2022). Effective prevention and management require a multimodal approach, patient engagement, and trained professionals (McNamara et al. 2012). Acute pain services address these needs by providing direct patient care and supporting disciplines to deliver safe and effective pain management (Stamer et al. 2020). Key acute pain service roles include educating patients, advising on pharmacological and non‐pharmacological interventions, and training nurses and physicians (Rawal 2016). Acute pain service thus enhances perioperative care by integrating education, interdisciplinary collaboration, and tailored pain management strategies. In the majority of pain services in western countries, the specialised pain nurse (in the Netherlands: pain nurse consultant) has a leading role, under the supervision of an anaesthesiologist (nurse‐based‐anaesthesiologist‐supervised model) (Chen et al. 2025).

### 1.1 Background

The first acute pain service in the Netherlands was introduced in the mid‐1990s, and most hospitals had implemented such a service, following the nurse‐anaesthesiologist‐supervised model, within 15 years (van Boekel et al. 2015). National guidelines, quality criteria, committees established by the government, the Dutch Health Care Inspectorate, and professional associations supported this process (van Boekel et al. 2015; van den Heuvel et al. 2024).

Transitional Pain Services, a newer concept, focuses on the entire patient journey. This includes preoperative identification of patients at risk for severe or chronic pain, followed by personalised perioperative guidance to prevent chronic pain and opioid dependence (Katz et al. 2015; Tiippana et al. 2016). Recently, some Dutch hospitals have started implementing transitional pain services, aligning with international developments (Admiraal et al. 2023; Klimke et al. 2024).

Although acute pain service and transitional pain service are well documented (Al‐Saidi et al. 2023; Macres et al. 2023; Stamer et al. 2020), further evidence is needed to standardise their implementation and ensure consistent quality. There is variation in team composition, roles, responsibilities, and availability (Hoogervorst‐Schilp et al. 2016; Kailainathan et al. 2018). This is particularly true of specialist nurses. Although they are trained holistically and play an important role in pain assessment, monitoring pain and opioid use, and providing pain education, there is insufficient literature to determine exactly which competencies are required at which level and which nurse should fulfil which role.

A recent review of four European countries highlighted disparities in perioperative pain management and noted a lack of up‐to‐date information for the Netherlands (van den Heuvel et al. 2024).

### 1.2 Aim

This study aims to provide an up‐to‐date overview of the organisation, roles, and responsibilities of the acute pain service and transitional pain service in the Netherlands, as a basis for strategies to improve pain services and enhance the quality of perioperative pain management for surgical patients.

## 2 Methods

### 2.1 Design

This cross‐sectional study was conducted between 7 May 2024 and 24 July 2024, using an online questionnaire distributed to participants via an electronic link to ensure accessibility and consistency.

### 2.2 Study Setting and Sampling

All Dutch hospitals performing inpatient surgery were eligible. Data from the National Institute for Public Health and the Environment (RIVM) were used to identify hospitals and their locations, then verified for inpatient surgery. Contact persons for acute pain service and/or transitional pain service were requested, prioritising nurse pain consultants or pain nurses because of their well‐known overview knowledge of these pain services. If no pain service was available, a contact person from the department responsible for pain management was invited to respond.

### 2.3 Inclusion and/or Exclusion Criteria

All Dutch hospitals performing inpatient surgery were included.

### 2.4 Instrument with Validity and Reliability/Data Source

A questionnaire, based on previous research (van Boekel et al. 2015) and updated with current evidence (Admiraal et al. 2023; Al‐Saidi et al. 2023), was piloted by pain experts to assess face validity and content. Definitions of acute pain service and transitional pain service were included to ensure respondents had a consistent understanding. Definition of complete acute pain service: A specialised hospital service managing acute postoperative pain to improve comfort, recovery, and prevent complications (Stamer et al. 2020).

Definition of complete transitional pain service: A service identifying and managing patients at risk for severe acute or chronic pain, supporting perioperative care, and preventing opioid misuse (Katz et al. 2015; Tiippana et al. 2016). Partial acute pain service or partial transitional pain service was defined when services met only parts of the definition.

The questionnaire included sections on hospital demographics, availability of acute pain service and transitional pain service, and details on organisation, staffing, roles, training, patient‐related and non‐patient‐related activities, and financial aspects. Questions were primarily closed‐ended, with optional comment fields for additional input.

### 2.5 Data Collection

Participants received an information letter and consented before accessing the online questionnaire via Castor EDC. Reminder emails and phone calls were used to address non‐responses. Non‐responders were asked whether an acute pain service or transitional pain service existed in their hospital to ensure accurate prevalence data.

### 2.6 Data Analysis

All data were processed descriptively, including missing data. Questionnaire data were described as frequencies accompanied by percentages when variables were nominal. Medians and interquartile ranges were reported for continuous variables. Data on hospital characteristics and availability of acute pain services and transitional pain services were analysed using data from all respondents. Characteristics of acute pain services and transitional pain services were analysed using only the data from respondents who indicate that they have an acute pain service and/or transitional pain service as defined before. Data were analysed using descriptive statistics with SPSS (IBM Corp. 2005. IBM SPSS Statistics for Windows, version 29.0.2.0 Armonk, NY: IBM Corp.).

### 2.7 Ethical Considerations

Ethical approval was granted by the Institutional Review Board of Radboud University Medical Centre (2024‐17207 METC Oost‐Nederland). Written informed consent was obtained from all participants, and data were de‐identified before processing.

## 3 Results

### 3.1 Characteristics of the Sample: Participating Hospitals

A total of 92 hospitals with inpatient surgery were initially approached to participate in the study, including three children's hospitals. Of these, six hospitals did not consent, leaving 86 eligible institutions. Of the consenting hospitals, nine did not respond to further requests to participate. Ultimately, 77 (83.7%) hospitals completed the study questionnaire, making their data eligible for analysis. Questionnaires were completed by various medical professionals, including 32 (41.6%) nurse pain consultants, 11 (14.3%) recovery room nurses, 8 (10.4%) acute pain service nurses, 8 (10.4%) nurse practitioners, 7 (9.1%) nurse anaesthetists, 5 (6.5%) anaesthesiologists, and 2 (2.6%) pain physicians.

### 3.2 Availability of Acute Pain Service and Transitional Pain Service

Of the 77 responding hospitals, 59 (76.6%) reported having a complete acute pain service (Table 1). Nearly all university medical centres and most general district hospitals with medical education had a complete acute pain service. Hospitals with a partial acute pain service focused on patient care, excluding non‐patient‐related activities such as evaluating pain outcomes. Five hospitals (6.5%) reported having a complete transitional pain service, including three teaching hospitals, one children's teaching hospital, and one district general hospital. Hospitals with a partial transitional pain service indicated they provided postoperative opioid advice but no follow‐up after discharge. Among the 15 non‐responding hospitals, 11 (78.6%) had an acute pain service, leading to a total acute pain service coverage (complete and partial) of 89.1% across the Netherlands. None of the non‐responding hospitals reported having a transitional pain service. Non‐participation was mainly due to staff shortages or time constraints.

**TABLE 1:** Availability of complete acute pain services and transitional pain services, and partial acute pain services and transitional pain services in hospitals in the Netherlands.

	Number of hospitals (*N*, %)	APS complete (*N*, %)	APS partial (*N*, %)	TPS complete (*N*, %)	TPS partial (*N*, %)
*Type of hospital*					
	77	59 (76.6)	12 (15.6)	5 (6.5)	10 (13.0)
University medical centre	10 (13.0)	9 (90.0)	1 (10.0)	5 (50.0)	2 (20.0)
District general hospital with medical education and training	61 (79.2)	47 (77.0)	11 (18.1)	0 (0.0)	7 (11.5)
District general hospital	3 (3.9)	2 (66.7)	0 (0.0)	0 (0.0)	1 (33.3)
Independent treatment centre	2 (2.6)	0 (0.0)	0 (0.0)	0 (0.0)	0 (0.0)
Missing	1	1			
*Size of hospital: number of beds*					
	61	46 (75.4)	10 (16.4)	3 (4.9)	9 (14.8)
< 200	17 (27.9)	9 (52.9)	3 (17.6)	0 (0.0)	4 (23.5)
200–500	25 (41.0)	21 (84.0)	4 (16.0)	2 (66.7)	2 (8.0)
> 500	19 (31.1)	16 (84.2)	3 (15.8)	1 (33.3)	3 (15.8)
Missing	16	13	2	2	1

*Note:* Missing data: not all respondents filled in the type of hospital or the number of beds. Abbreviations: APS, acute pain service; TPS, transitional pain service.

Of the 15 hospitals with a transitional pain service, 11 (73.3%) fully integrated acute pain service and transitional pain service, with the same staff managing both. Three hospitals had separate but collaborative transitional pain services, while one had a separately organised acute pain service and a transitional pain service that did not collaborate. All pain services were available for both adult and paediatric patients, excluding acute pain services at children's hospitals.

For consistency, hereafter only data from hospitals with complete acute pain services and/or transitional pain services were analysed.

### 3.3 Organisation Acute Pain Services and Transitional Pain Services

#### 3.3.1 Acute Pain Services

Most acute pain services (76.3%) operate within the anaesthesiology department, with others under the pain clinic (8.4%) or other departments (15.3%), such as recovery or operating rooms. Funding comes primarily from the anaesthesiology department (39.0%), hospital funds (27.1%), the anaesthesia fee (22.0%), or other sources. Anaesthesiologists (64.4%) and pain physicians (66.1%) are mostly responsible for pain management, followed by nurse practitioners (8.5%) and others.

#### 3.3.2 Transitional Pain Services

Transitional pain services are often part of the anaesthesiology department (80.0%) and/or pain clinic (60.0%), primarily funded by anaesthesiology (60%). Pain physicians hold ultimate responsibility, sometimes shared with nurse practitioners or physician assistants.

### 3.4 Working Hours of Pain Services

Acute pain services were available during daytime hours on all weekdays (33.9%) or all working days (23.7%), in combination with limited service outside office hours. Other acute pain services operated only during weekdays (13.6%), all days of the week (5.1%), or on a 24/7 basis (10.2%). Data were missing for 6.8% of teams, while another 6.8% reported alternative schedules, such as daytime availability on weekdays and Saturday mornings. Transitional pain services were primarily available during daytime on working days (40.0%) or weekdays with reduced service outside office hours (40.0%). Only 20% of transitional pain services were integrated into outpatient pain management.

Among acute pain services, 40 (67.8%) considered working hours sufficient, whereas 15 (25.4%) disagreed, citing concurrent duties in recovery or operating theatres. Data from 4 (6.8%) respondents were missing. Respondents highlighted a need for improved weekend availability to monitor patients undergoing Friday surgeries. Limited out‐of‐hours pain services were often covered by on‐call anaesthesiologists, who were not consistently available due to other clinical responsibilities.

### 3.5 Composition and Staffing of the Team

The median FTE for acute pain services (*N* = 59) was 2.1 [IQR: 1.2–3.5]. Anaesthesiologists (45.8%), pain physicians (50.8%), nurse pain consultants (64.4%), recovery nurses (50.8%), and nurse anaesthetists (42.4%) were most commonly part of acute pain services, while surgeons were not involved (Table 2). The median FTE for transitional pain services (*N* = 5) was 1.0 [IQR: 1.0–1.0]. Anaesthesiologists/pain specialists (80.0%), nurses (80.0%), and nurse pain consultants (60.0%) were included in most transitional pain services, and psychologists, physiotherapists, and rehabilitation specialists were absent. Respondents reported understaffing and unfilled vacancies. FTE numbers of acute pain services may correlate with hospital size: small hospitals (0–200 beds, *N* = 17) had a median FTE of 2.0 [IQR: 0.6–4.0], medium hospitals (201–500 beds, *N* = 25) had 2.6 [IQR: 1.1–3.7], and large hospitals (> 500 beds, *N* = 19) had 2.8 [IQR: 1.2–4.4]. Data were unavailable for 16 hospitals. FTE for transitional pain services could not be calculated due to the small sample size.

**TABLE 2:** Number and FTE of professionals working in complete acute pain services (*N* = 59) and complete transitional pain services (*N* = 5) in the Netherlands.

Discipline	APS teams number median [IQR]	APS teams FTE median [IQR]	TPS teams number median [QR]	TPS teams FTE median [IQR]
Pain consultant (specialised nurse)	3.0 [2.0–5.0]	1.5 [1.0–2.5]	2.0 [ID]	1.3 [ID]
Pain consultant (specialised nurse anaesthetist)	2.0 [1.0–3.0]	1.0 [0.3–1.7]	0	0
Recovery room nurse (specialised nurse)	3.0 [1.5–5.5]	0.7 [0.3–1.0]	0	0
Intensive care nurse (specialised nurse)	1.0 [ID]	0.7 [ID]	0	0
Nurse practitioner	1.0 [1.0–1.75]	0.8 [0.8–1.0]	1 [1.0–1.0]	0.6 [0.6–0.6]
Nurse anaesthetist	4.0 [2.0–6.0]	0.8 [0.28–1.3]	0	0
Physician assistant	1.0 [1.0–1.0]	0.9 [ID]	1.0 [1.0–1.0]	0
Anaesthesiologist	7.5 [1.75–14.25]	1.3 [0.1–3.8]	5.0 [5.0–5.0]	1.0 [1.0–1.0]
Anaesthesiologist/pain physician	3.0 [1.0–5.0]	1.0 [0.1–1.5]	1.5 [0.9–11.0]	0.6 [ID]
Other	1.0 [ID]	0.6 [ID]	1.0 [ID]	1.0 [ID]

*Note:* ID = insufficient data: due to some low numbers not all IQRs were given; Other = For APS, nurses from different departments were indicated. For TPS, it was indicated that the psychologist or rehabilitation physician could be consulted. Abbreviations: APS, acute pain service; FTE, full‐time equivalent; TPS, transitional pain service.

### 3.6 Education of Acute Pain Services and Transitional Pain Services

In Table 3 is shown that almost all staff received some additional training in pain management. Half of the acute pain service staff (57.6%), primarily nurses, have completed post‐graduate training in pain management. Transitional pain service staff predominantly undertook internal training programmes.

**TABLE 3:** Education in pain followed by professionals working in complete acute pain services (*N* = 59) and complete transitional pain services (*N* = 5) in the Netherlands.

Education	APS teams (*N*, %)	TPS teams (*N*, %)
Internal training	16 (27.1)	3 (60.0)
Training EQF4 Nurse assistant in pain and pain management (4 days)	3 (5.1)	0 (0.0)
Post graduate programme EQF6 nurse Acute pain service (7 days)	29 (49.2)	0 (0.0)
Post graduate programme EQF6 pain nurse Belgium (2 years)	6 (10.2)	1 (20.0)
Post graduate programme EQF6 pain consultant Netherlands(2 years)	34 (57.6)	1 (20.0)
Post graduate programme medical pain consultant Netherlands	6 (10.2)	0 (0.0)
Post graduate programme medical pain consultant other countries	1 (1.7)	0 (0.0)
Other	7 (11.9)	2 (40.0)
No education	4 (6.8)	0 (0.0)

*Note:* Several options could be filled in for this question. Abbreviations: APS, acute pain service; EQF, European Qualification Framework (used as an indication of educational level); TPS, transitional pain service.

### 3.7 Tasks and Patient‐Related Activities Acute Pain Services

Acute pain services regularly attend surgical patients with advanced analgesic techniques such as PCIA pumps (93.2%), epidural catheters (93.2%), locoregional catheters (83.1%), or intrathecal catheters (39.0%). They are also consulted for acute non‐surgical pain (83.1%) and oncological pain (62.7%), or when ward nurses lack experience with specific pain regimens (62.7%). Some teams visit all patients in pain, while others focus only on surgical cases with advanced techniques, citing staffing constraints or lack of training for non‐surgical patients. Respondents expressed interest in integrating non‐pharmacological and lifestyle interventions into standard acute pain service care.

Additionally, respondents also mentioned that administrative tasks consume a lot of time that could preferably be spent with the patient, and that their work could be supported by the use of health technology, for example, patients monitoring their own pain scores in a pain app.

### 3.8 Tasks and Patient‐Related Activities of Transitional Pain Services

Transitional pain service tasks include identifying patients at risk of severe pain or opioid use pre‐ (60%) and postoperatively (80%), and tapering opioids preoperatively (20%) and after discharge (100%) (Table 4). Transitional pain services focus on patients with difficult‐to‐treat pain, high‐risk surgeries, neuropathic pain risk, pre‐operative opioid use, and psychological factors like anxiety or catastrophising (Table 5). Contact with patients was conducted entirely by telephone (100%) and/or in outpatient clinics (60.0%). One respondent indicated visiting patients on the ward during admission.

**TABLE 4:** Tasks of complete transitional pain services (*N* = 5) in the Netherlands.

	*N* (%)
Preoperative identification of patients at risk^a^	3 (60.0)
Postoperative identification of patients at risk^a^	4 (80.0)
Preoperative guidance in reducing and stopping opioids	1 (20.0)
Preoperative interdisciplinary consultation about the patient	3 (60.0)
Preoperative pain education to patients	3 (80.0)
Periodic follow‐up after discharge (e.g., every week)	5 (100.0)
Calling the patient once after discharge	1 (20.0)
Contacting general practitioner (GP) about pain management	5 (100)
After the patient is discharged, contacting the main practitioner (surgeon)/GP about pain management	2 (40.0)
Post‐discharge, interdisciplinary consultation about the patient	2 (40.0)
Post‐discharge, providing guidance on tapering and discontinuing opioids	5 (100)
Post‐discharge, screening for the development of neuropathic pain	4 (80.0)
Post‐discharge, providing guidance on reducing and discontinuing opioids, facilitated by the pharmacy	2 (40.0)
Post‐discharge management of (repeat) prescriptions for pain medication	3 (60.0)
Post‐discharge, referring patients to specialised clinics, such as pain management centres or addiction treatment facilities	4 (80.0)
Other	2 (40.0)

*Note:* Other = Among others: Providing nonpharmacological interventions, handing out a schedule for tapering opioids.

^a^ Type of surgery where a percentage of patients are known to develop chronic postoperative pain and/or long‐term opioid use.

**TABLE 5:** Patients visited by complete transitional pain services (*N* = 5) in the Netherlands.

	(*N*, %)
Risk of difficult‐to‐treat acute postoperative pain	5 (100.0)
Risk of chronic postoperative pain	5 (100.0)
High risk surgery chronic postoperative pain^a^	3 (60.0)
Risk of neuropathic pain	4 (80.0)
Risk of long‐term opioid use	5 (100.0)
Psychological risk factors	3 (60.0)
Pre‐operative opioid usage	4 (80.0)
Pre‐operative chronic pain	5 (100.0)
Usage of relatively large numbers of opioids during admission	4 (80.0)
Patients with pain during admission that is difficult to control	3 (60.0)
Other	0 (0.0)

^a^ Type of surgery where a percentage of patients are known to develop chronic postoperative pain.

Many respondents noted a strong demand for transitional pain service development, with many teams in early implementation stages. Respondents commented that a lack of knowledge about transitional pain service organisation causes delays in patient registration and undermines the preventive approach of transitional pain service support.

### 3.9 Characteristics of Pain Services: Quality and Safety of Pain Care

Pain services also focus on coordinating quality and safety in pain care, particularly through professional training. Respondents mentioned a lack of knowledge among doctors and nurses about pain in general, recognising pain, and the possibilities of pharmacological and non‐pharmacological treatment of pain, including opioid tapering. They also named as a problem the lack of awareness about the consequences of pain, the risk of pain becoming chronic, and long‐term opioid use.

Acute pain services provide pain management training in 89.8% of Dutch hospitals. Additional training for nurses is offered in 79.7% of hospitals upon request, and for physicians in 35.6%. Regular training is provided to nurses in 45.8% of hospitals and to physicians in 13.6%. The integration of pain education into the basic professional training of nurses and nurse specialists by pain services is (11.9%) and for physicians and physician assistants (3.4%). Similarly, pain services are involved in pain education in the advanced training programmes for nurses (15.3%) and physicians (3.4%). Respondents noted a knowledge gap among physicians and nurses, especially in recognising severe pain, prescribing opioids, and understanding the benefits of non‐pharmacological interventions.

Furthermore, almost 60% of acute pain services evaluate the multidisciplinary pain management policy in the hospital and provide active verbal feedback on pain data regarding outcomes at the departmental level. Data is also used to advise on the development of treatment plans, protocols, and/or procedures related to pain and pain management. Additionally, almost 70% of acute pain services monitor the responsible use of opioids in the hospital.

### 3.10 Research

Eighteen out of 77 hospitals (23.4%) are actively engaged in scientific research on postoperative pain. Furthermore, 2 respondents reported conducting scientific research in perioperative pain management, with both studies having a principal investigator from their own transitional pain service. Respondents remarked that they would like to share more knowledge, experience, and research outcome at a national level.

## 4 Discussion

This research provides an overview of the organisation, tasks, and responsibilities of Dutch acute pain services and transitional pain services since the initial survey about Dutch acute pain services in 2015. Almost all of the hospitals surveyed had an acute pain service; few hospitals reported having a fully established transitional pain service. Additionally, a large portion of hospitals with a transitional pain service reported that it was integrated with the acute pain service.

The prevalence of complete and partial acute pain services in the Netherlands is nearly ninety percent (89.1%), comparable to that in the UK (90%) and Germany (81%), and higher than most other western countries (50%–90%) (Erlenwein et al. 2016; Rockett et al. 2017; Tawfic et al. 2021). Considerable variation exists in organisational structure, staffing, and performance of acute pain services within and between western countries (Erlenwein et al. 2021; Rockett et al. 2017; Tawfic et al. 2021), and greater clarity in the definitions used is required for meaningful comparisons to be made. Nevertheless, there are also similarities. For instance, specialised pain nurses often provide patient education and training for nurses and physicians (Sonneborn and Miller 2021). Still, there is a lack of evidence regarding acute pain services and the role of pain nurses worldwide.

In addition, some hospitals have smaller teams of specialised nurse pain consultants, while others rely on larger teams that include all recovery nurses (van den Heuvel et al. 2024). Literature shows that dedicated and specialised teams contribute to better quality of care (Al‐Saidi et al. 2023; Cassarino et al. 2021; Rawal 2005; Shapiro et al. 2004). Whether dedicated pain service teams influence pain care outcomes requires further research.

Acute pain services in the Netherlands are organised according to the nurse‐anaesthesiologist‐supervised model (Chen et al. 2025). Although anaesthesiologists or pain specialists are ultimately responsible, it is mainly pain nurse consultants who advise nurses and ward physicians. This role of pain nurse consultants is fragile because, although the roles, responsibilities, and powers of a pain nurse are often defined within the institution, there is no legal regulation at a national level, as is the case for nurse practitioners and physician assistants. A bottleneck mentioned in this context is that anaesthesiologists or pain physicians are not always available in time for consultations, especially outside office hours.

The prevalence of transitional pain services remains limited, with Canada, the USA, Finland, and the UK being pioneers (Katz et al. 2015; Tiippana et al. 2016). In the Netherlands, transitional pain services often consist only of a pain physician and a nurse pain consultant or nurse practitioner; psychologists and physiotherapists are not structurally part of the team (Klimke et al. 2024; Mikhaeil et al. 2020; Tiippana et al. 2016). This can be explained by the fully multidisciplinary chronic pain care system in the Netherlands, with transitional pain service seen as a precursor.

A challenge is the lack of transparency regarding staffing (FTE) within the acute pain service. Although anaesthesiologists contribute to acute pain service duties, there is not always a specific FTE allocation for their involvement. Pain services wish to expand acute pain service and transitional pain service working hours and tasks, but face difficulties due to limited data for informed negotiations on realistic FTE requirements. Despite proven benefits and cost‐effectiveness (Al‐Saidi et al. 2023; Bojic et al. 2023; Mikhaeil et al. 2020), justifying further resource allocation remains challenging. Additionally, pain services can reduce overall healthcare costs, while the costs of pain services are mainly covered by hospitals (Huang et al. 2016; Mikhaeil et al. 2020).

A significant proportion of acute pain service and transitional pain service staff have received additional training in pain management, similar to other Western countries (Erlenwein et al. 2021; Rockett et al. 2017; Sonneborn and Miller 2021). However, there are differences in the type, duration, and level of training, such as two‐year postgraduate training, e‐learning modules, and internal training, whereas in other countries specialised training is mainly at postgraduate and master's level (Erlenwein et al. 2021; Sonneborn and Miller 2021). International associations recommend advanced training for acute pain service and transitional pain service staff (EFIC 2013; O'Brien et al. 2017).

Based on the low number of TPS teams, it is difficult to make a statement on this, but it would be interesting to investigate the relationship between level and type of training followed and the extent to which and how a TPS is implemented. This is because in the Dutch training for pain nurse consultants, implementation, project management and content information on transitional pain service is a regular part.

Respondents were concerned about the knowledge of ward physicians and nurses in pain management, for example, in recognising severe pain, prescribing opioids, and the possibilities of non‐pharmacological interventions. Research (McCabe et al. 2023) has shown that less than half of nurses lack adequate knowledge about pain. Despite this knowledge gap (EFIC 2013; McNamara et al. 2012; Prempreet et al. 2022), basics of pain management are not fully included in the curricula of most initial education for healthcare professionals (EFIC 2013). As a result, acute pain services spend a lot of time retraining physicians and nurses in these basics. Research shows that education in pain management basics leads to better pain management and appropriate opioid prescribing (Durbhakula et al. 2024). Topics such as pain assessment, understanding pain behaviours, patient misconceptions about pain and medication, pharmacological and non‐pharmacological pain management were identified as necessary basic knowledge for nurses (McCabe et al. 2023).

Acute pain services see almost all patients with complex treatment techniques and a large proportion with acute non‐surgical and oncological pain. International research and guideline criteria for acute pain services emphasise the inclusion of patients with complex acute pain or severe comorbidity (Popping et al. 2008; Stamer et al. 2020). Acute pain services report that this is not always possible due to staffing and time constraints, as recognised in previous research (Chang et al. 2010; Powell and Davies 2012).

Respondents also note that treatment often only consists of pharmacological interventions and value non‐pharmacological interventions as a preferable addition. Literature states that postoperative pain management is often approached from a biomedical perspective only, whereas a biopsychosocial approach, including non‐pharmacological interventions, is more effective (Akire and Bayraktar 2024). These interventions (such as relaxation exercises and music) take little time, have no side effects, can be delivered by ward nurses, and are used by patients after discharge (Small and Laycock 2020).

Transitional pain services in the Netherlands are in early stages of implementation, making comparisons with the US and Canada difficult (Klimke et al. 2024). The optimal transitional pain service model for the Dutch healthcare system remains unclear and requires further study. Few pain services engage in research on perioperative and postoperative pain, despite the need for knowledge and expertise. Workforce and financial limitations hinder research efforts. Collaborative research and knowledge sharing are crucial, with professional associations, research institutes, and educational institutions playing key roles (O'Brien et al. 2017). Specifically for nurses, research could contribute to a better understanding of the different roles, levels of intervention and advice, and required competencies, for example with regard to the use of non‐pharmacological interventions at basic and advanced levels. Recent insights, such as the importance of more holistic approaches and the empowerment of patients to prevent and reduce pain, align with the profession of (pain) nursing (Benhamou and Forget 2025). However, there is a knowledge gap in research regarding specific roles and competencies of nurses in pain management, in particular for specialised pain nurses.

### 4.1 Strengths and Limitations

This study provides an overview of acute pain services and transitional pain services in the Netherlands, highlighting their organisation, roles, and responsibilities. The high response rate and diverse representation of Dutch hospitals enhance generalisability. A key strength is the rigorously developed and piloted questionnaire, refined with input from pain experts. However, limitations include the self‐reported nature of the data. Observational research or follow‐up interviews could offer more objective insights. Voluntary participation may have led to over‐representation of hospitals with advanced pain services.

Since this study focuses on pain services in the Netherlands, its findings may not apply to countries with different healthcare policies or pain management practices. Future research could expand to international comparisons, facilitating global benchmarks for acute pain service and transitional pain service (Duncan et al. 2014).

### 4.2 Recommendations

To develop, standardise and expand pain services, more research is needed on optimal pain service models in the Netherlands. More evidence on issues such as transparency of staffing (FTE), appropriate resource allocation and required staff competencies can support the quality of pain services. To improve pain management in surgical patients, more evidence is needed on the organisation and implementation of a biopsychosocial model, including tailored pharmacological and non‐pharmacological interventions. Finally, it would be advisable to conduct research into pain services for specific target groups. For example, with pain in children and neonates, as it requires even greater customisation and specific expertise.

To promote research and knowledge sharing, collaboration between hospitals, research institutes and professional associations needs to be encouraged to improve perioperative and postoperative pain research.

### 4.3 Implications

The findings of this study suggest that, despite the widespread presence of acute pain services and the growth of transitional pain services, the quality of pain services remains a concern. The present study provides evidence of the organisational structure, roles and responsibilities in the Netherlands, and can support pain nurse consultants, anaesthetists, pain physicians and hospital managers in implementing and innovating pain services in hospitals.

It is recommended that educational institutions be encouraged to develop their pain curriculum, with the findings of this research serving as a foundation for such initiatives.

Similar studies should be conducted in other Western countries so that comparative analyses can be used to further develop pain services. Also recommended is follow‐up research on more detailed information on the role of nurses and pain nurses in postoperative pain management.

## 5 Conclusion

This study highlights the organisation, tasks, and responsibilities of acute pain services and transitional pain services in the Netherlands, including challenges such as staffing transparency and the recent implementation of transitional pain services. Future efforts should focus on optimising staffing, expanding research on transitional pain services, and establishing a national framework for basic pain management education to improve effective and consistent pain care.

## Disclosure

Statistics: There is a statistician/epidemiologist on the author team: Rianne van Boekel. The author(s) affirm that the methods used in the data analyses are suitably applied to their data within their study design and context, and the statistical findings have been implemented and interpreted correctly. The author(s) agree to take responsibility for ensuring that the choice of statistical approach is appropriate and is conducted and interpreted correctly as a condition to submit to the Journal.

## Ethics Statement

Ethical approval was granted by the Institutional Review Board of Radboud University Medical Centre (2024–17,207 METC Oost‐Nederland). Written informed consent was obtained from all participants, and data were de‐identified before processing.

## Conflicts of Interest

The authors declare no conflicts of interest.

## Data Availability

The data that support the findings of this study are available on request from the corresponding author. The data are not publicly available due to privacy or ethical restrictions.
